# Advancing multidisciplinary management of pediatric hyperinflammatory disorders

**DOI:** 10.3389/fped.2025.1553861

**Published:** 2025-04-30

**Authors:** Francesco La Torre, Giovanni Meliota, Adele Civino, Angelo Campanozzi, Valerio Cecinati, Enrico Rosati, Emanuela Sacco, Nicola Santoro, Ugo Vairo, Fabio Cardinale

**Affiliations:** ^1^ Department of Pediatrics, Giovanni XXIII Pediatric Hospital, University of Bari, Bari, Italy; ^2^ Pediatric Cardiology, Giovanni XXIII Pediatric Hospital, Bari, Italy; ^3^ Pediatric Rheumatology and Immunology Unit, “Vito Fazzi” Hospital, Lecce, Italy; ^4^ Pediatric Unit, Department of Medical and Surgical Sciences, University of Foggia, Foggia, Italy; ^5^ Pediatric and Pediatric Oncohematology Unit, Santissima Annunziata Hospital, Taranto, Italy; ^6^ Neonatology and Intensive Care Unit, “Vito Fazzi” Hospital, Lecce, Italy; ^7^ Pediatric Unit, Fondazione IRCCS Casa Sollievo della Sofferenza, Foggia, Italy; ^8^ Pediatric and Oncohematology Unit, Azienda Ospedaliero Universitaria “Policlinico Consorziale”, Bari, Italy

**Keywords:** hyperinflammation, still disease, Kawasaki disease, MIS-C multisystem inflammatory syndrome, recurrent pericarditis, biologics, IL-1, IL-6

## Abstract

Pediatric hyperinflammatory diseases, including Still's disease, Kawasaki disease (KD), multisystem inflammatory syndrome in children (MIS-C), and recurrent pericarditis (RP), represent a spectrum of conditions characterized by immune dysregulation and systemic inflammation. Each disorder exhibits distinct pathophysiological mechanisms and clinical features, yet their overlapping presentations often pose diagnostic challenges. Early and accurate differentiation is critical to mitigate complications such as macrophage activation syndrome (MAS), coronary artery aneurysms, and myocardial dysfunction. This narrative review explores the pathophysiology, diagnostic criteria, and management of these conditions, emphasizing the utility of advanced biomarkers, imaging modalities, and genetic testing. For Still's disease, the review highlights the transformative role of biologic therapies targeting IL-1 and IL-6 in reducing systemic inflammation and improving outcomes. In KD, timely administration of intravenous immunoglobulin (IVIG) and combination with high-dose steroids in high-risk patients is pivotal for preventing coronary complications. MIS-C, associated with SARS-CoV-2 infection, requires tailored immunomodulatory approaches, including corticosteroids and biologics, to address severe hyperinflammation and multiorgan involvement. RP management prioritizes NSAIDs, colchicine, and IL-1 inhibitors to reduce recurrence and corticosteroid dependence. The review advocates for a multidisciplinary approach, integrating standardized diagnostic algorithms and disease-specific expertise to optimize patient care. Future research directions include the identification of predictive biomarkers, exploration of novel therapeutic targets, and development of evidence-based treatment protocols to enhance long-term outcomes in pediatric inflammatory diseases.

## 1 Introduction

Pediatric autoinflammatory and hyperinflammatory conditions are characterized by dysregulated immune responses resulting in excessive systemic inflammation (1). These diseases encompass a spectrum of disorders, including Still's disease, Kawasaki disease (KD), multisystem inflammatory syndrome in children (MIS-C), and recurrent pericarditis (RP). While autoinflammation primarily involves innate immune dysregulation, hyperinflammation often arises from exaggerated activation of both innate and adaptive immunity, frequently triggered by infections, malignancies, or genetic predispositions (1). Despite their shared features, such as fever, elevated acute-phase reactants, and multiorgan involvement, each condition presents unique pathophysiological mechanisms, clinical manifestations, and therapeutic challenges.

Still's disease is a polygenic autoinflammatory disease characterized by systemic inflammation, presenting as intermittent fever, rash, and arthritis (2). Cytokine dysregulation, particularly involving IL-1 and IL-6, plays a central role in its pathogenesis, and complications such as macrophage activation syndrome (MAS) and interstitial lung disease (ILD) can be observed (2–6). Diagnosis relies on clinical evaluation and the exclusion of conditions such as leukemia, lymphoma, and other autoimmune or inflammatory disorders with overlapping features (7–10). Recent advances, including the introduction of IL-1 and IL-6 inhibitors, have improved outcomes, with IL-1 antagonists now considered first-line therapy (2, 8, 11–13).

KD is a systemic vasculitis predominantly affecting children under 5 years of age (14, 15). It presents with persistent fever, conjunctival hyperemia, mucocutaneous inflammation, and cervical lymphadenopathy (16, 17). Coronary artery involvement is the hallmark complication, potentially leading to aneurysm formation if untreated (14, 17–19). The disease is driven by vascular endothelial injury mediated by immune complex deposition and cytokine dysregulation (20–22).

MIS-C is a severe hyperinflammatory syndrome temporally related to SARS-CoV-2 infection (23). It is driven by a cytokine storm associated with excessive IL-1β, IL-6 and TNF-α activity, leading to systemic vasculitis and myocardial dysfunction (23). It is characterized by fever and multiorgan dysfunction, commonly involving cardiac, gastrointestinal, renal, and neurological systems, and laboratory evidence of inflammation (24, 25). Although MIS-C shares features with KD, such as fever and mucocutaneous involvement, it differs in the frequency of myocarditis and gastrointestinal symptoms (26, 27).

RP involves IL-1 dysregulation, leading to persistent repeated episodes of pericardial inflammation (28, 29). It is often idiopathic or secondary to autoinflammatory syndromes such as familial Mediterranean fever or Still's disease (30–32). Key clinical features include chest pain, pericardial effusion, and electrocardiographic changes (28–30).

The nonspecific and overlapping early presentations of these conditions often delay diagnosis, increasing the risk of severe complications. Distinguishing these conditions from infectious, oncological, or autoimmune diseases requires advanced biomarkers, imaging techniques, and genetic testing (33–36). In KD, incomplete presentations can mimic viral infections, requiring echocardiographic evaluation for coronary abnormalities (37). MIS-C shares clinical features with KD and sepsis, necessitating the use of biomarkers like N-Terminal Pro–B-Type Natriuretic Peptide (NT-proBNP) and cardiac troponins to confirm cardiac involvement (38). Differentiating Still's disease from malignancies or infections may involve bone marrow aspiration and infectious disease panels, while distinguishing RP from other inflammatory diseases often requires cardiac magnetic resonance (MRI)and computed tomography (CT) (32, 39).

Given the complexity of these diseases, a multidisciplinary approach is essential to optimize patient outcomes and address disease-specific challenges. This includes standardized diagnostic algorithms combining clinical, laboratory, and imaging data, as well as targeted use of biologics to reduce systemic inflammation and prevent complications. Training pediatric subspecialists, such as rheumatologists and cardiologists, is critical to ensuring disease-specific expertise. Efforts such as those in the Apulia region in southern Italy aim to develop integrated diagnostic and therapeutic care pathways tailored to regional healthcare systems.

This review aims to provide a comprehensive exploration of pediatric Still's disease, KD, MIS-C, and RP, with a focus on their distinct pathophysiological mechanisms, clinical presentations, and evidence-based management strategies. The authors seek to propose diagnostic and therapeutic algorithms for treatment escalation in these conditions, highlighting indications for the early initiation of biological therapies.

## 2 Methods

The authors formed a multidisciplinary working group within the Apulia region, comprising pediatric rheumatologists, immunologists, cardiologists, oncologists, and other specialists actively engaged in clinical care. The primary aim was to develop standardized diagnostic and therapeutic recommendations for managing hyperinflammatory conditions based on the best available evidence.

The authors conducted an online search for articles on pediatric Still's disease, KD, MIS-C, and RP. Relevant articles were retrieved from PubMed using various combinations of the search terms: “pediatric Still's disease” or “systemic juvenile idiopathic arthritis” OR “Kawasaki disease” OR “multisystem inflammatory syndrome in children” OR “pediatric recurrent pericarditis” AND “diagnosis” OR “therapy.” Articles were initially screened by title and abstract for relevance, followed by a full-text review. To broaden the scope of this narrative review, they included studies of all designs. Additionally, the authors screened the reference lists of selected articles to identify further relevant literature. Previously published review articles underwent full-text screening to identify gaps in the existing research and provide a more comprehensive discussion of this topic.

## 3 Still's disease

Systemic juvenile idiopathic arthritis (sJIA) is a subtype of juvenile idiopathic arthritis (JIA), accounting for 10%–15% of all JIA cases (2, 40–44). Recently, the EULAR/PRES recommendation defined systhemic juvenile idiopathic arthritis and adult-onset Still's disease (AOSD) as the same disease that should be designated by the same unique name, Still's disease (formerly called sJIA/AOSD) (8). Pediatric Still's disease affects males and females equally, with a peak incidence between 1 and 5 years of age (44, 45).

The hallmark features of Still's disease include persistent fevers lasting at least two weeks, an evanescent salmon-pink rash, arthralgia or arthritis, lymphadenopathy, hepatosplenomegaly, and serositis (Table 1) (2, 8, 46, 47). The course of the disease is variable, with good outcomes in patients with a monophasic course, other patients developing chronic, unremitting arthritis, and others experiencing a polyphasic course (2, 50–52). Despite extensive research, no biomarkers reliably predict which patients will experience a chronic, refractory disease course (45, 53–57).

**Table 1:** Diagnostic criteria for SJIA based on ILAR, CARRA and PRINTO classifications.

Criteria set	Items	Minimal requirements
ILAR criteria (2001) (7)	Major criteria: (1) Age ≤16 years (2) Arthritis persists for ≥6 weeks. (3) Daily fever for ≥2 weeks. Minor criteria: (4) Evanescent erythematous rash. (5) Generalized lymphadenopathy (6) Hepatomegaly or splenomegaly. (7) Serositis.	All major criteria plus ≥1 of minor criteria.
CARRA (2012) (48)	Major criteria: (1) Age ≤16 years (2) Fever ≥2 weeks (3) Arthritis in one or more joints (duration not specified). Minor criteria: (4) Evanescent erythematous rash. (5) Generalized lymphadenopathy (6) Hepatomegaly or splenomegaly. (7) Serositis.	All major criteria plus ≥1 of minor criteria
PRINTO Criteria (2023) (49)	Major criteria: (1) Age ≤18 years (2) Fever for ≥3 consecutive days recurring over ≥2 weeks. (3) Evanescent erythematous rash. (4) Arthritis Minor criteria: (5) Generalized lymphadenopathy (6) Hepatomegaly or splenomegaly. (7) Arthralgia without arthritis (8) Serositis (9) Neutrophilic leukocytosis ≥15,000/mm^3^).	Items (1) and (2) plus another major criteria or two minor criteria
Additional notes	Excludes conditions like infections, malignancies (via bone marrow biopsy if needed), and other autoimmune diseases.	
Clinical indicators	- Key cytokines: IL-1 and IL-6 elevation. - Elevated acute-phase reactants (e.g., ferritin, CRP). - Biomarkers such as serum calprotectin, IL-18 and S100 proteins assist in differentiating sJIA from overlapping syndromes like KD and MIS-C.	

CRP, C-reactive protein; IL-1, interleukin-1; IL-6, interleukin-6; IL-18, Interleukin-18; SJIA, systhemic juvenile idiopathic arthritis; KD, Kawasaki disease; MIS-C, multisystem inflammatory syndrome in children.

Diagnosis relies on clinical criteria and the exclusion of other diseases (8). The International League of Associations for Rheumatology (ILAR) defines sJIA as arthritis lasting at least 6 weeks, with quotidian fever for 2 weeks and at least one of the following: evanescent erythematous rash, lymphadenopathy, hepatosplenomegaly, or serositis (Table 1) (7). The CARRA criteria differ from the ILAR criteria only in that the duration of arthritis is not specified (Table 1) (48). Newer PRINTO criteria exclude arthritis as an essential diagnostic feature, emphasizing the systemic nature of sJIA (49): fever for at least 3 consecutive days and reoccurring over a duration of at least 2 weeks and accompanied by one major criteria or two minor criteria. Major criteria are evanescent (non-fixed) erythematous rash and/or arthritis (49). Minor criteria are generalized lymph node enlargement and/or hepatomegaly and/or splenomegaly; serositis; arthralgia lasting 2 weeks or longer (in the absence of arthritis); and leukocytosis (≥15,000/mm3) with neutrophilia (Table 1) (49).

Differential diagnosis is challenging due to overlapping features with infections, oncological conditions, and autoimmune disorders (9, 10, 58–60). Biomarkers such as calprotectin (MRP8/14), IL-18, and S100 proteins help differentiate Still's disease from other conditions, including KD and MIS-C (8, 33, 35, 36).

The pathogenesis of Still's disease involves a biphasic immune response (2). The acute phase is driven by innate immune dysregulation, with IL-1 and IL-6 playing central roles in fever, rash, and systemic inflammation (2). Elevated ferritin, indicative of macrophage activation, is a common marker during this phase (2). Over time, IL-1 activation can stimulate adaptive immunity, transitioning to chronic arthritis characterized by synovial hyperplasia and cartilage destruction (2).

Historically, treatment relied on corticosteroids, particularly for systemic-phase disease. Advances in biologic DMARDs (bDMARDs) have transformed Still's disease management (12, 61, 62), with IL-1 inhibitors (e.g., anakinra, canakinumab) and IL-6 inhibitors (e.g., tocilizumab) achieving rapid systemic control, and reducing corticosteroid dependence (11, 63–69). In particular, anakinra has demonstrated significant efficacy in Still's disease, particularly in controlling inflammation and preventing disease progression (70, 71). Studies support its use as first-line monotherapy in new-onset, steroid-naïve Still's disease patients, showing rapid disease control and favorable long-term outcomes (72). Long-term safety data from the Pharmachild Registry indicate that anakinra is generally well tolerated, with a manageable safety profile, including a low incidence of severe adverse events (73). Similarly, canakinumab has proven effective in treating Still's disease, with studies demonstrating sustained remission and reduced flare rates (67, 74, 75). Canakinumab has also been investigated as a first-line steroid-free monotherapy, showing promising results in achieving inactive disease (76). Regarding safety, long-term studies indicate that canakinumab is well tolerated, with a favorable risk-benefit profile and a low incidence of severe infections or treatment-related complications (77). Tocilizumab is effective in Still's disease, with better outcomes when initiated early (78). Long-term studies confirm its sustained efficacy in controlling systemic and articular symptoms (79). While generally well tolerated, infections remain a concern (79).

Early initiation of biologics, ideally within 3 months of disease onset, maximizes efficacy and minimizes glucocorticoid exposure (11, 13, 70, 80). Notably, polymorphisms in the *IL1RN* gene, encoding the IL-1 receptor antagonist, may influence susceptibility and therapeutic responses to IL-1 inhibitors (81).

Corticosteroids remain essential for managing life-threatening systemic features, with pulse methylprednisolone or oral prednisone commonly employed (82).

Emerging treatments, including JAK inhibitors (e.g., ruxolitinib) (83), emapalumab (interferon-*γ* inhibitor) (84), and IL-18-targeted therapies (85), are under investigation for refractory cases and MAS-related hyperinflammation.

The sJIA Disease Activity Score (sJADAS) integrates systemic features, including joint count, global assessments, and acute-phase reactants [e.g., C-reactive protein (CRP), erythrocyte sedimentation rate (ESR)], to guide treatment and monitor disease progression (86, 87).

Complications include macrophage activation syndrome (MAS) and interstitial lung disease (ILD) (88, 89).

MAS usually refers to a secondary form of hemophagocytic lymphohistiocytosis (HLH) associated with autoimmunity, although there are other causes of secondary HLH, such as infections and malignancy (90). MAS is a life-threatening cytokine storm syndrome that affects 10% of pediatric Still's disease patients, with subclinical features in up to 40% (3, 90, 91). It presents with persistent, high-grade fever, hepatosplenomegaly, lymphadenopathy, and hemorrhagic manifestations (90) and carries an 8% mortality rate (92, 93).

Diagnostic criteria include ferritin >684 ng/ml and at least two additional abnormalities such as low platelet count (≤181 × 10^9^/L), hypertriglyceridemia (>156 mg/dl), elevated aspartate aminotransferase (>48 U/L), or hypofibrinogenemia (≤360 mg/dl) (90) (Table 2). Early identification of MAS through careful monitoring of laboratory biomarkers in high-risk patients who show clinical exacerbation despite appropriate treatment is fundamental to preventing irreversible organ damage due to hyper-cytokinemia (90, 92, 95).

**Table 2:** Diagnostic criteria for HLH/MAS.

Criteria set	Items	Minimal requirements
HLH-2004 (Primary HLH) (94)	(1) Molecular diagnosis consistent with HLH (2) Fever (3) Splenomegaly (4) Cytopenias (affecting ≥2 of 3 lineages in the peripheral blood): (5) Hemoglobin <90 g/L (in infants <4 weeks: hemoglobin <100 g/L) (6) Platelets <100 × 10^9^/L (7) Neutrophils <1.0 × 10^9^/L (8) Hypertriglyceridemia and/or Hypofibrinogenemia: Fasting triglycerides ≥3.0 mmol/L (i.e., ≥265 mg/dl) Fibrinogen ≤1.5 g/L (9) Hemophagocytosis in bone marrow or spleen or lymph nodes (10) No evidence of malignancy New diagnostic criteria: (11) Low or absent NK-cell activity (according to a local laboratoryreference) (12) Ferritin ≥500 mg/L (13) Soluble CD25 (i.e., soluble IL-2 receptor) ≥2,400 U/ml	Item 1 or ≥5 of items 2–13
MAS (sJIA-associated) (2016) (81)	(1) Ferritin ≥684 ng/ml: (2) Platelets ≤181 × 10^9^/L. (3) AST >48 U/L. (4) Triglycerides >156 mg/dl. (5) Fibrinogen ≤360 mg/dl.	Item 1 plus ≥2 of items 2–5
HLH/MAS diagnostic Scores	- HScore (adults) (89): Quantifies HLH likelihood based on ferritin, triglycerides, hepatomegaly and splenomegaly, and fever. - MH Score (children) (88): Quantifies HLH likelihood based on age at onset, neutrophil count, fibrinogen, splenomegaly, plateletcount, and hemoglobin. Differentiates primary and secondary HLH.	N.A.
Clinical indicators	- Persistent fever >38.5 °C. - Hemorrhagic manifestations. - Hepatosplenomegaly. -Lymphadenopathy	N.A.

N.A., Not applicable; HLH, Hemophagocytic lymphohistiocystosis.

Distinguishing MAS from Still's disease flares or sepsis-like syndromes is challenging (92). In active Still's disease, laboratory findings typically reveal elevated platelet counts, fibrinogen levels, and ESR, whereas MAS is characterized by a decrease in these parameters, though CRP levels increase in both conditions (96, 97). Diagnostic and classification tools for HLH/MAS include the HScore for adults and the MH Score for pediatric patients, the latter of which aids in distinguishing between primary and secondary HLH (Table 2) (98, 99).

Genetic susceptibility to HLH/MAS has been identified, with macrophage activation triggered by inflammatory processes, malignancies, or viral infections, such as Epstyein-Barr (EBV), cytomegalovirus (CMV), SARS-CoV-2, adenovirus, HSV, and RSV (100–102). Due to overlapping clinical features, experts recommend genetic testing for primary HLH in cases of recurrent MAS in pediatric Still's disease patients (101).

High-dose pulse intravenous methylprednisolone (30 mg/kg/day for 3–5 days) remains first-line therapy for MAS (101). Intravenous immunoglobulin (IVIG) is also frequently added to methylprednisolone as part of the initial combination therapy, particularly in pediatric cases where MAS commonly coexists with infections (101).

Anti-IL-1 therapies, such as anakinra (4–12 mg/kg/day), have shown efficacy in second-line treatment of refractory MAS (101, 103–106). Tocilizumab, an anti-IL-6 agent, may also be considered for refractory MAS, particularly when anakinra is unavailable, though its efficacy appears somewhat lower (106). Emapalumab is emerging as a promising treatment for MAS leading to improvement of clinical and laboratory parameters with a favorable safety profile (84). To monitor disease activity and guide treatment, serial evaluation of leukocyte and platelet counts, aminotransferase and ferritin levels, and coagulation parameters are recommended (107, 108).

Emerging evidence links Still's diseaseto ILD, a rare but severe complication whose risk factors include early age of onset, high disease activity, extensive rash, MAS, trisomy 21, lymphopenia, eosinophilia, and biologic infusion reactions (4–6). Screening with pulmonary function tests and imaging (high-resolution computed tomography) is recommended for high-risk patients to enable early intervention (5).

In addition, cardiovascular ailments can occur in Still's disease patients, primarily manifesting as pericarditis, occurring in about 10% of Still's disease patients, with echocardiographic signs detected in over 30% of cases. However, it typically resolves with effective disease control (109, 110).

## 4 Kawasaki disease

KD is a systemic febrile vasculitis primarily affecting children under 5 years old (14, 15) and is the leading cause of acquired heart disease in developed countries (14, 15, 111). Global incidence varies widely, with Japan reporting the highest rates (260/100,000 children in 2012) compared to 25/100,000 in the USA and 10–15/100,000 in Europe (112–116). Boys are more frequently affected, and the disease is usually diagnosed before 5 years of age (14, 15, 115).

Diagnosis requires fever lasting at least 5 days and at least four of the following symptoms: rash, conjunctival injection, oral mucosal changes (e.g., strawberry tongue), extremity changes (e.g., erythema or swelling), and cervical lymphadenopathy (17, 117).

Incomplete or atypical KD, characterized by fewer than four clinical criteria, often occurs in infants under six months or children over five years, delaying diagnosis and increasing the risk of coronary complications (17, 117, 118). The recently updated statement of the American Heart Association for the management of Kawasaki disease confirms that for suspected incomplete KD is mandatory the presence of prolonged unexplained fever (at least 7 days for infants) and 2–5 clinical criteria associated with laboratory or echocardiographic findings as coronary involvement (*Z*-score of LAD CA or RCA ≥2.5) or elevated CRP (≥3 mg/dl), and/or ESR (≥40 mm/h), associated with at least three of the following items: anemia for age, leukocytosis (≥15,000 mmc), thrombocytosis (450,000 mmc), elevated ALT, leukocyturia (≥10/hpf), and hypoalbuminemia (≤3 g) (17, 117).

A recent proteomic and genetic study identified four distinct KD phenotypes: hepatic involvement, high neutrophil counts with cardiogenic shock risk, lymphoglandular expression, and early-onset KD associated with aneurysms (119). These findings highlight the need for individualized risk and treatment strategies.

KD Shock Syndrome (KDSS),a severe form associated with myocarditis, hypotension, and increased IVIG resistance, occurs in younger patients (mean age: 3.7 years) and has a mortality rate of 6.8% (120, 121).

Elevated cytokines, including IL-1β, TNF-α, and IL-17, drive systemic inflammation and endothelial activation, leading to vascular damage (20–22). Genetic studies highlight polymorphisms in immune regulatory genes, reinforcing the role of genetic predisposition (122).

Prompt treatment within 10 days of symptom onset significantly reduces coronary complications (117). IVIG (2 g/kg) is the cornerstone therapy, resolving inflammation in the great majority of cases, alongside high-dose aspirin (30–50 mg/kg/day, tapered to 3–5 mg/kg/day after fever resolution) (15, 117, 123). For IVIG-resistant cases, which represent approximately 10%–20% of cases, adjunctive therapies include corticosteroids and biologics (117, 124–126). Methylprednisolone pulses (30 mg/kg/day for three days) reduce coronary artery lesions (108), often in combination with IVIG (127). Biologic agents such as infliximab (anti-TNF) and anakinra are increasingly used for severe or refractory cases, with anakinra demonstrating efficacy in preventing coronary complications and controlling hyperinflammation (128–136).

Long-term outcomes depend on timely treatment. While most treated children recover without sequelae, 5%–8% of treated and 25% of untreated patients develop coronary aneurysms (14, 19, 34), with 50%–80% of giant aneurysms persisting up to 10 years after diagnosis (137). Coronary aneurysms are associated with inflammatory cell infiltration and endothelial remodeling, contributing to thrombosis and ischemia (15, 34). Advances in imaging and treatment have improved prognosis, leading to approximately 90% long-term survival (138). Echocardiography is crucial for detecting coronary artery involvement, and the novel Coronary Ectasia Diagnostic Index (CEDi) has shown high sensitivity and specificity for early identification (34). A CEDi value of ≥0.41 (left coronary) or ≥0.39 (right coronary) at admission is diagnostic, with 97% normalization within eight weeks (34).

MAS occurs in approximately 1% of KD cases (139, 140), though subclinical forms may be underdiagnosed. While rare, MAS in KD may be underdiagnosed, particularly in its subclinical forms. Diagnosis of MAS in KD is complicated by the overlap of symptoms with severe KD forms, such as IVIG-resistant KD and KDSS. Both conditions share features such as prolonged fever, thrombocytopenia, and hyperferritinemia, making it difficult to differentiate them clinically. Several studies suggest that the 2016 sJIA-MAS classification criteria (90), which emphasize hyperferritinemia (>684 ng/ml) and laboratory abnormalities such as thrombocytopenia, hypofibrinogenemia, elevated AST, and hypertriglyceridemia, may be more suitable for identifying MAS in KD compared to HLH-2009 criteria (139, 141). In a cohort of 719 KD patients, 1.1% were diagnosed with MAS using Ravelli's criteria (90), but only 0.42% met the HLH-2009 criteria, suggesting that the latter has low sensitivity for KD-associated MAS (139). A retrospective study of KD patients complicated by MAS found that affected individuals frequently exhibit fever > 5 days, oral mucosal changes, hepatomegaly, bilateral conjunctival injection, rash and extremity changes, thrombocytopenia, and elevated inflammatory markers such as ferritin and lactate dehydrogenase (139). Hemophagocytosis in bone marrow aspirates was observed in some but not all cases, reinforcing the notion that this feature is not required for diagnosis (139).

Early recognition and prompt treatment of MAS in KD are crucial to prevent severe complications, including multi-organ failure. The treatment of MAS in patients with KD is similar to that in Still's disease, and anakinra has shown success, reinforcing the potential role of IL-1 blockade in managing MAS in KD (142).

## 5 Multisystem inflammatory syndrome in children

MIS-C is a hyperinflammatory condition related to SARS-CoV-2 infection, typically manifesting 2–6 weeks after infection with a median presentation age of 8–9 years (23, 143).

MIS-C disproportionately affects Afro-Caribbean children and presents with severe systemic inflammation distinct from but overlapping with KD and other hyperinflammatory syndromes (26, 27, 59, 144–146).

The global incidence varies, with a rate of 0.11 cases per million person-months in the USA in 2023 (147) and a cumulative incidence of 3.27 per 100,000 children in southern Italy (148).

MIS-C is characterized by persistent intractable fever, gastrointestinal symptoms, coagulopathy, shock, mucocutaneous lesions, lymphadenopathy, and cardiovascular complications (24, 25, 149).

MIS-C diagnosis follows criteria from the World Health Organization (WHO). These criteria are characterized by the presence of fever ≥3 days in children or adolescents under 19 years old associated with elevation of inflammatory markers (PCR, ESR or procalcitonin), SARS-CoV-2 exposure, no obvious microbial cause of inflammation and two of the following clinical features: (1) Rash or bilateral non-purulent conjunctivitis or mucocutaneous inflammation signs; (2) hypotension or shock; features of myocardial dysfunction, pericarditis, valvulitis, or coronary abnormalities (including echocardiographic findings or elevated troponin/NT-proBNP); (3) evidence of coagulopathy [by prothrombin time [PT], partial thromboplastin time [PTT], elevated D-Dimers] and/or (4) acute gastrointestinal problems (diarrhea, vomiting, or abdominal pain (148, 150). Besides these, clinical findings include elevated D-dimer, NT-proBNP, cardiac troponins, and cardiac involvement, in particular left ventricular dysfunction (LVEF <50%), which is a prognostic feature present in 30%–60% of patients (26, 96, 151–156). LVEF, however, is reversible and typically normalizes at discharge (157–159). Left ventricular dysfunction can initially be assessed through basic electrocardiographic screening (160). This can be further supported by speckle-tracking echocardiographic evaluation, which measures left ventricular myocardial strain (161, 162), or by cardiac MRI, which can detect edema and fibrotic replacement with limited tissue involvement during the acute phase of the condition, providing additional insights into disease activity (160, 163).

The use of high-sensitivity troponins allows for the detection of nonspecific myocardial involvement, which may not necessarily be associated with myocarditis (164, 165).

Coronary artery aneurysms are less frequent in MIS-C compared to KD and the occurrence of complications, such as giant aneurysms, is rare (27, 166).

A study by Aydin et al. (96) reported the presence of serositis in 46.2% of MIS-C cases, with no statistically significant difference between the groups. Yashuara et al. (159) reported that mild mitral regurgitation, observed at onset in 50% of MIS-C cases, may serve as a soft marker indicative of early disease. Importantly, when appropriate treatment and supportive care are provided during the acute phase, cardiac involvement does not seem to adversely affect long-term prognosis (24).

Neurological symptoms such as headaches and encephalopathy further highlight the syndrome's systemic nature (167).

Unlike KD, MIS-C frequently presents with thrombocytopenia, reflecting coagulopathy (146).

Differential diagnosis of MIS-C is based on older age at onset (27, 168), pronounced myocardial dysfunction, and unique biomarker profiles such as elevated NT-proBNP, BNP, and cardiac troponins (27, 96, 151–153). Additionally, patients display higher fibrinogen levels and lymphopenia and relatively modest ferritin elevations compared to KD (96).

While MAS may develop during MIS-C, it generally follows a milder course with favorable outcomes compared to MAS in Still's disease (169).

Pathophysiologically, MIS-C involves a cytokine storm driven by elevated TNF-α, IFN-γ, IL-1β, and IL-6, causing widespread organ damage (23). Transcriptomic studies suggest MIS-C and KD lie on an inflammatory spectrum, with MIS-C representing a more severe manifestation driven by IL-15/IL-15RA dysregulation (26). Neutralizing autoantibodies targeting the interleukin-1 receptor antagonist (IL-1Ra) may account for increased IL-1β-signaling in MIS-C, suggesting a mechanistic role for IL-1 dysregulation in MIS-C pathogenesis (170). This discovery highlights potential therapeutic interventions, such as the use of anakinra (170). Importantly, these autoantibodies were observed less frequently in children with KD or other inflammatory disorders, emphasizing their specificity to MIS-C and enhancing our understanding of its unique pathophysiology (170).

Treatment of MIS-C targets systemic inflammation, myocardial dysfunction, and coagulopathy. Early intervention is fundamental to avoid life-threatening complications (171, 172). IVIG (2 g/kg) is usually the starting therapy, often combined with corticosteroids [intravenous (IV), 1–2 mg/kg/day] to enhance efficacy (25, 153, 173). At present, the use of a second dose of IVIG is not recommended due to the associated risks of hemolytic anemia and cardiovascular volume overload, particularly in patients with compromised left ventricular function (153). High-dose methylprednisolone (IV, 30 mg/kg/day for 3 days) is employed in life-threatening moderate to severe cases to control cytokine storm and prevent shock (25, 173). For refractory cases or in cases of myocarditis and in life-threatening severe patients, IL-1 inhibitor anakinra [>4 mg/kg/day, IV or subcutaneous (SC)] is effective in mitigating severe inflammation and preventing cardiovascular complications (25, 153, 173–175). Anticoagulation therapy with enoxaparin is recommended for thrombotic risk or significant coronary artery involvement or for patients with moderate or severe LV dysfunction (EF <35%) to prevent thromboembolic events (25, 173). Aspirin (ASA) therapy at an anti-aggregating dose (3–5 mg/kg/day) is usually recommended in patients who are not considered or after the acute phase as a replacement for anticoagulant therapy and continued for at least 8 weeks from the onset of fever. A percentage varying between 30% and 80% of MIS-C patients require intensive care due to hemodynamic instability or multiorgan dysfunction (171, 176). Despite severe acute presentations, most patients recover cardiac function within weeks, though long-term echocardiographic and MRI follow-ups are recommended for persistent abnormalities (152, 177). Early initiation of immunomodulatory therapies has reduced MIS-C mortality to 1.7%–2.6% (148, 171, 173, 176).

## 6 Recurrent pericarditis

Recurrent pericarditis occurs in approximately 22%–36% of pediatric patients after an initial episode of acute pericarditis (178, 179), with higher recurrence rates when colchicine is not included in initial treatment or when initial treatment included corticosteroids (180, 181). Although uncommon in children, RP is increasingly recognized due to improved diagnostic techniques and heightened awareness.

RP is characterized by episodes of sharp, pleuritic chest pain that worsens in a supine position and improves when sitting upright or leaning forward (30, 181). Additional findings include pericardial friction rub, present in 30%–40% of cases, and pericardial effusion, typically small to moderate in size but occasionally large (30, 181, 182). Systemic symptoms, such as fever, malaise, and elevated inflammatory markers, often accompany flares, mimicking systemic autoinflammatory syndromes (30).

Diagnosis requires at least one recurrence following a symptom-free interval of 4–6 weeks after an initial episode (30). Clinical criteria include two or more features of pericarditis: typical chest pain, pericardial effusion (imaged by echocardiography), pericardial friction rub, or electrocardiographic changes, such as PR segment depression or widespread concave ST elevation (30). Elevated CRP and ESR further support the diagnosis (30). Imaging techniques such as echocardiography are essential for evaluating effusion and ruling out alternative causes of chest pain (181).

Pediatric RP is idiopathic in most cases but can be linked to systemic diseases, such as Still's disease, or monogenic autoinflammatory syndromes, such as familial Mediterranean fever (FMF) and tumor necrosis factor receptor-associated periodic syndrome (TRAPS) in 6%–10% of patients (30).

Pathophysiologically, RP involves dysregulated inflammation driven by overexpression of IL-1 and other proinflammatory cytokines, leading to immune-mediated pericardial injury and fibrosis (28, 29). Chronic inflammasome activation may increase the risk of complications, such as constrictive pericarditis or cardiac tamponade (28, 29), though these remain rare in children (30).

Treatment of RP aims to alleviate symptoms, prevent recurrences, and minimize corticosteroid dependency. NSAIDs are first-line therapy (30, 181), with high doses of ibuprofen (30–50 mg/kg/day in three doses). Aspirin and indomethacin, though effective, are less favored in children due to hepatic (ASA) and neurological (indomethacin) side effects (181, 183–185). Indomethacin, though effective, is less favored in children due to neurological side effects (172, 174). As mentioned before, adding colchicine to NSAIDs significantly reduces recurrence rates (30, 180, 181, 185), and treatment should be continued for at least 6 months after recurrence (183). Recommended dosages are weight-based: 0.5 mg/day for children and adolescents under 70 kg and 1 mg/day for those over 70 kg (180, 181, 186).

Corticosteroids are reserved for cases refractory to NSAIDs and colchicine, but they should be added to aspirin/NSAIDs and colchicine and not used as standalone therapy (181). Low-dose regimens (0.2–0.5 mg/kg/day prednisone) with gradual tapering are preferred to minimize relapse risk and side effects (183). Notably, prolonged corticosteroid use increases recurrence rates and may interfere with colchicine efficacy (183).

Anakinra is highly effective for colchicine-resistant or corticosteroid-dependent RP (184, 187, 188). In particular, the study by Caorsi et al. (184) involving 57 pediatric patients showed that anakinra significantly reduced relapses in patients previously treated with NSAIDs (43%), NSAIDs + colchicine (17%), steroids (4%), NSAIDs + steroids (24%), and NSAIDs + colchicine + steroids (12%), even after steroid withdrawal. Early use of anakinra in colchicine-resistant cases before corticosteroid initiation facilitated faster disease control and reduced side effects (184). Importantly, the study highlighted that the first-line treatments (NSAIDs and colchicine) were underdosed, and even high doses of steroids failed to achieve complete control of pericarditis flares in 33% of patients (184). Furthermore, 94% of treated patients exhibited steroid dependence withdrawal (184). Notably, 10 patients from the study showed a prompt response to IL-1 blockers without prior steroid treatment withdrawal (184).

Typical doses of anakinra for RP treatment range from 1 to 2 mg/kg/day, administered subcutaneously (102, 180, 183, 184, 187). Treatment is generally continued for at least six months, with careful tapering to assess recurrence risk. In rare cases of anakinra intolerance, high-dose canakinumab may be considered, though results have been less consistent (184).

With appropriate treatment, the long-term prognosis of RP is favorable, though up to 36% of pediatric patients experience multiple relapses (178, 179, 185, 189). Early introduction of colchicine and IL-1 inhibitors is crucial to reducing recurrence risk (28, 30, 184). Chronic inflammation can lead to rare complications like constrictive pericarditis or tamponade, necessitating close monitoring with cardiac MRI in high-risk patients (182). Recurrent symptoms and corticosteroid side effects can adversely affect quality of life, underscoring the importance of steroid-sparing therapies to mitigate these impacts.

## 7 Expert opinion

### 7.1 Recommendations for a multidisciplinary care pathway

Managing pediatric hyperinflammatory diseases requires a multidisciplinary approach tailored to the specific condition and individual patient needs. Integrating evidence-based guidelines with clinical expertise is critical to achieving optimal outcomes.

For diagnostic workup, we recommend a standardized panel of first-level tests, including:

- Basic bloodwork: complete blood count, electrolytes, urea, creatinine, and blood glucose.
- Inflammatory markers: ESR, CRP, ferritin, and fibrinogen.
- Liver and coagulation Panels: transaminases, albumin, PT, PTT, international normalized ratio (INR).
- Infectious workup: viral multiplex swabs for common pathogens and antibody tests for EBV, CMV, and adenovirus and parvovirus B19.
- Protein and urine analysis.

Initial imaging includes chest x-ray, electrocardiography (ECG), and abdominal ultrasonography to assess systemic involvement. Echocardiography, while essential for cardiovascular assessment, is considered a second-line test reserved for patients with suspected cardiac complications.

For cases with inconclusive first-line results or specific clinical suspicion, second-line tests are recommended. These include:

- Infectious disease workup: tests for HIV, hepatitis B and C (HBV, HCV), Leishmania, and SARS-CoV-2.
- Advanced EBV Testing: If EBV antibodies are positive, quantitative polymerase chain reaction (PCR) testing is advised to assess viral load.

For patients with hyperinflammation or suspected MAS, advanced diagnostic panels are essential for accurate assessment and timely management. Key tests include lactate dehydrogenase (LDH) and triglycerides, which help identify hyperinflammatory states. In cases meeting MAS criteria, such as thrombocytopenia and elevated ferritin (>648 ng/ml), centralized sample analysis at specialized centers is strongly recommended to ensure standardized and reliable results. Second-level investigations, including perforin expression, interleukin-18 (IL-18), and CXCL-9 levels, should also be conducted at these specialized centers to maintain consistency and diagnostic accuracy (35, 190–193). Such advanced testing plays a critical role in guiding therapy for complex cases.

Effective management of MAS requires a multidisciplinary approach with frequent clinical reassessments, particularly in rapidly evolving cases. Centralized diagnostic workflows not only enhance accuracy but also facilitate cohesive decision-making among specialists.

### 7.2 Disease-specific recommendations

#### 7.2.1 Still's disease

Differential diagnosis of Still's disease is critical to exclude oncological conditions. A bone marrow aspirate is recommended in cases of unexplained fever, bone pain, bicytopenia, or pancytopenia, and MRI bone marrow abnormalities, while minimally invasive lymph node biopsies may be necessary in cases of lymphadenopathy to guide therapy decisions. In cases of hyperinflammatory disease, the 2016 MAS Classification Criteria provide a first-line scoring system validated for sJIA, with good applicability to other inflammatory conditions (91, 194). The HLH-2004 criteria may also be used, particularly when there is suspicion of a genetic or familial predisposition (195) (Table 2). Referring to the 2022 EULAR recommendations, early MAS screening aids in identifying patients requiring further evaluation (101). Genetic testing, including clinical exome sequencing, should be considered in recurrent pediatric MAS cases.

**Figure 1:**
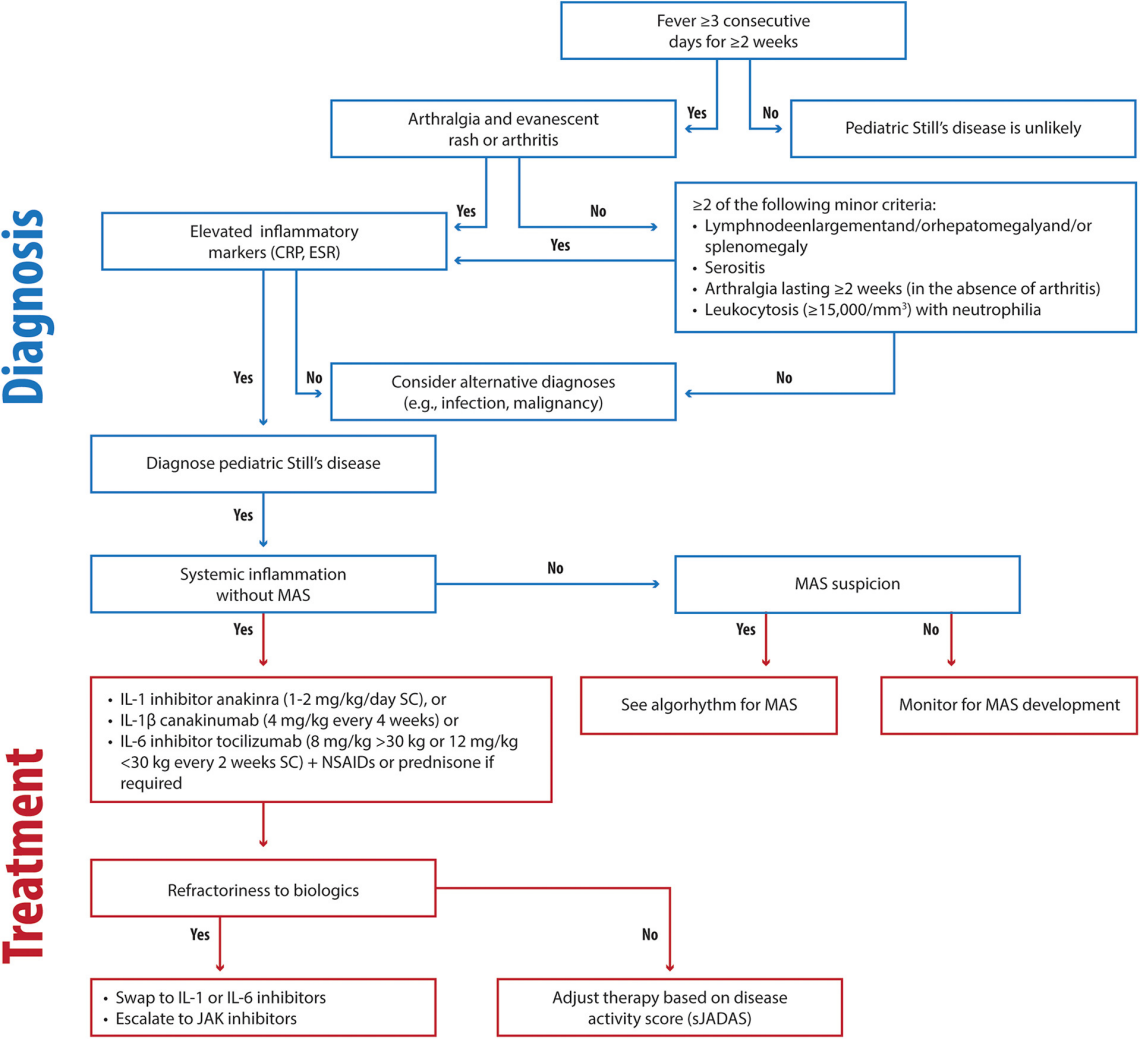
Clinical flowchart illustrating the diagnostic and therapeutic approach to Still's disease. CRP, C-reactive protein; ESR, Erythrocyte Sedimentation Rate; MAS, Macrophage Activation Syndrome; IV, intravenous; SC, subcutaneous.

A disease-specific, treat-to-target approach is essential for effective Still's disease management. Initiating IL-1 or IL-6 inhibitors as first-line therapy, even without corticosteroids, in agreement with the recent 2024 EULAR/PReS recommendations, is our recommended approach unless MAS is present (Figure 1) (8). In our experience and agreement with the literature, early use of biologics, such as short-acting anakinra during the differential diagnosis phase, improves long-term outcomes (2, 12, 13). Early intervention with first-line biologics, such as interleukin-1 inhibitors, during the initial inflammatory phase of Still's disease exploits a critical “window of opportunity” (2, 196). By targeting the innate immune system, this approach prevents its transition to adaptive immunity, thereby halting the progression from systemic to chronic articular Still's disease and potentially altering the disease trajectory (2, 196).

We are not in favor of the administration of corticosteroids as a standalone initial therapy; instead, NSAIDs may be beneficial for brief initial treatment to modulate the autoinflammatory process and potentially prevent progression to autoimmunity. Our clinical experience shows that rapid initiation of biologics combined with tailored steroid tapering effectively resolves systemic symptoms and improves outcomes, even in MAS-complicated cases.

In our opinion, treatment goals should include achieving ≥50% improvement within three months and remission or low disease activity within six months. Fever resolution in systemic Still's diseaseshould occur within one week of starting therapy. We recommend monitoring disease activity using the sJADAS (86, 87).

In the 2023 consensus paper by Shakoory et al. (101), a broad agreement on the treatment of HLH/MAS was not reached. However, baseline therapy with high-dose glucocorticoids and anakinra is considered a reasonable starting point. Notably, methylprednisolone has a stronger anti-inflammatory effect compared to other glucocorticoids and may be better tolerated at the hepatic level. The selection of a specific glucocorticoid often depends on the clinician's expertise. For HLH, international guidelines recommend dexamethasone due to its rapid onset of action, typically within 2–3 days.

The utility of cyclosporin A (CsA) in MAS treatment remains a subject of debate. While corticosteroid therapy alone may suffice in some cases, certain studies have demonstrated the efficacy of CsA in managing hyperinflammation (197, 198). Furthermore, fibrinogen administration in MAS should be approached cautiously, as it may induce liver damage; thus, careful evaluation of the patient's hepatic status is essential.

**Figure 2:**
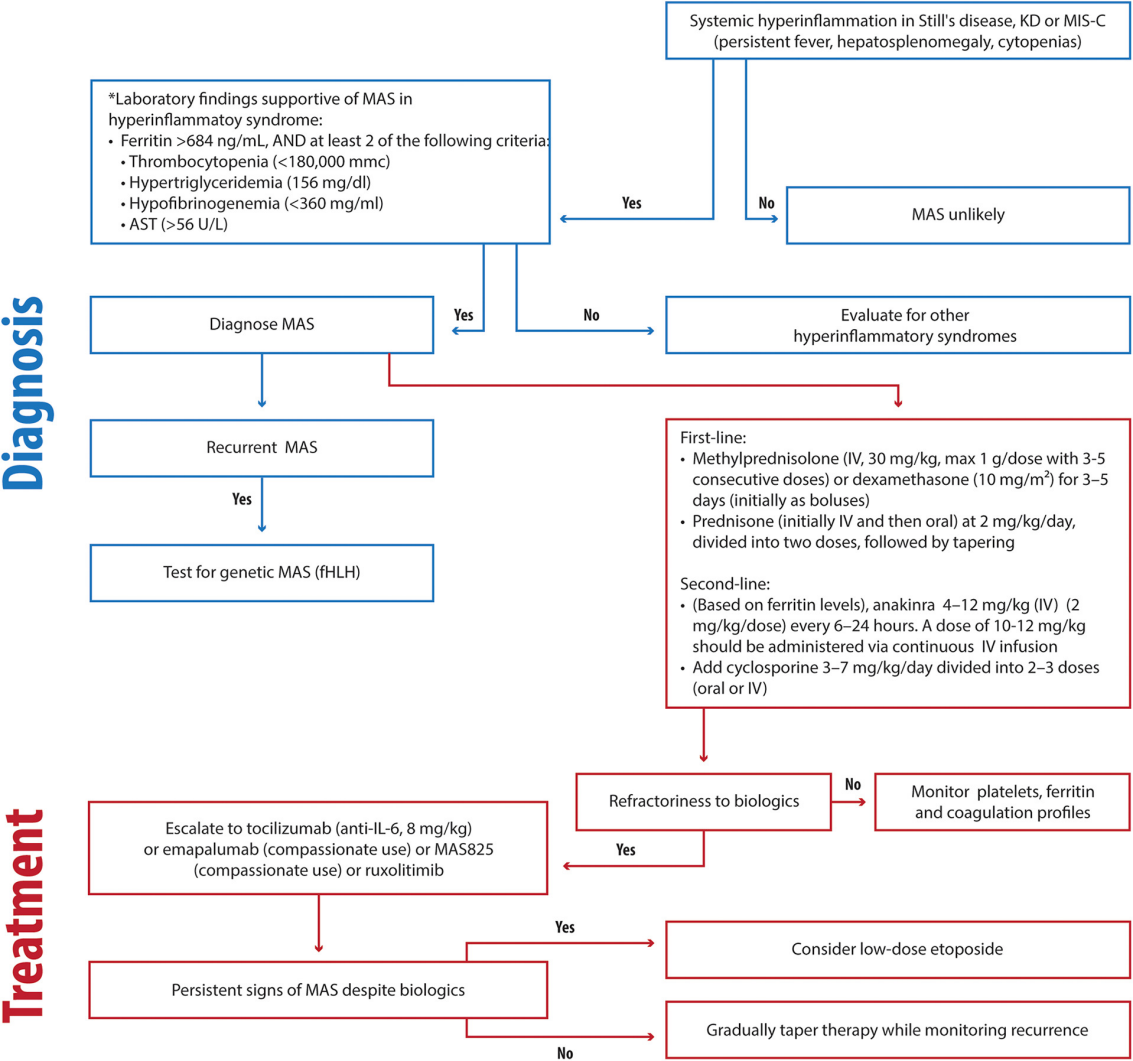
Clinical flowchart illustrating the diagnostic and therapeutic approach to MAS in hyperinflammatory disorders. KD, Kawasaki disease; MIS-C, Multisystem Inflammatory Syndrome in Children; fHLH, familial hemophagocytic lymphohistiocytosis; HLH, hemophagocytic lymphohistiocytosis; AST, Aspartate Aminotransferase.

We propose the following approach (Figure 2) for timely and effective MAS management in the absence of evidence of genetic disease:

- First-line therapy
  - Methylprednisolone (IV, 30 mg/kg, max 1 g/dose with 3–5 consecutive doses) or dexamethasone (10 mg/m^2^) for 3–5 days (initially as boluses)
  - prednisone (initially IV and then oral) at 2 mg/kg/day, divided into two doses, followed by tapering.
- Second-line therapy (based on ferritin levels)
  - anakinra 4–12 mg/kg/day intravenously (IV) (2–3 mg/kg/dose every 6–24 h). A dose of 12 mg/kg should be administered via continuous infusion.
  - ±cyclosporine 5–7 mg/kg/day divided into 2–3 doses (oral or IV).
- Advanced therapy
  - Ruxolitinib (Jack 1/2 inhibitors), 5 mg twice a day (BIS) (<10 kg); 5 mg BIS (<25 kg); 10 mg twice a day (BIS) (≥25 kg) (199).
  - Tocilizumab (anti-IL6), 8 mg/kg)
  - MAS825 (IL-1 and IL-18 inhibitor) 10 mg/kg (max 700 mg) biweekly (200)
  - Emapalumab (6 mg/kg/day on day 0, followed by 3 mg/kg every 3 days until day 15, and twice a week until day 28) (84).
  - Low-dose etoposide should be reserved as a last option.

Preference should be given to emapalumab or MAS825 when available. Ruxolitinib is typically used in the treatment of chronic hematological diseases due to its slow action.

#### 7.2.2 Kawasaki disease

**Figure 3:**
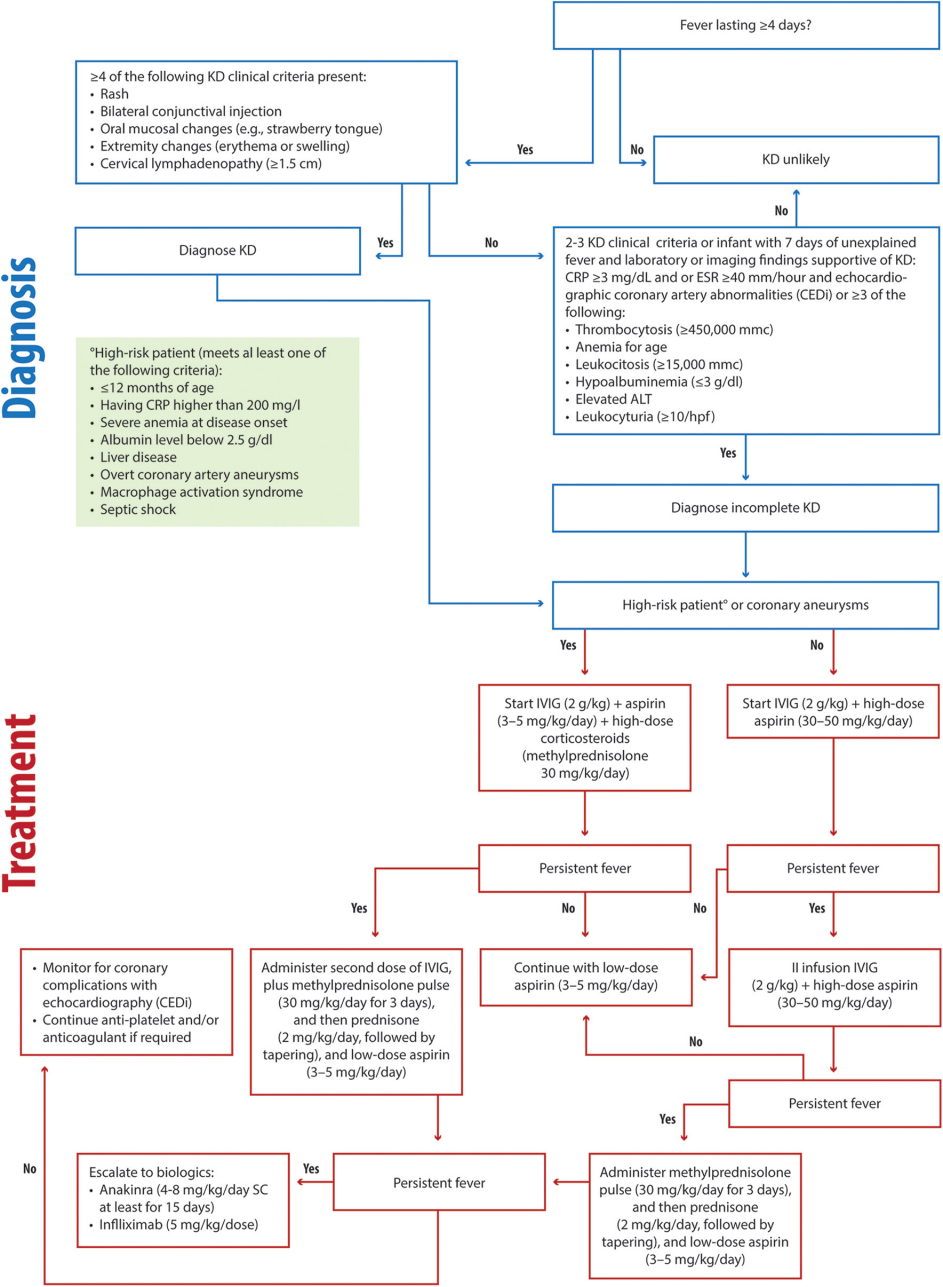
Clinical flowchart illustrating the diagnostic and therapeutic approach to KD. KD, Kawasaki disease RP, C-reactive protein; ESR, Erythrocyte Sedimentation Rate; ALT, alanine aminotransferase; IVIG, intravenous immunoglobulin.

KD remains a clinical diagnosis characterized by fever, unilateral lymphadenopathy, rash, bilateral nonexudative conjunctival injection, swelling and erythema of the hands and feet, and oropharyngeal findings, including strawberry tongue and erythematous lips (17, 117). The algorithm for diagnosing complete or incomplete KD, with supportive laboratory features, is shown in Figure 3.

Early administration of IVIG (2 g/kg) and aspirin (30–50 mg/kg/day) is critical for preventing coronary complications in KD patients, as recommended by current guidelines (17, 117) (Figure 3). For high-risk patients (children less than 12 months or children having CRP higher than 200 mg/L, severe anemia at disease onset, albumin level below 2.5 g/dl, liver disease, overt coronary artery aneurysms, macrophage activation syndrome or septic shock), we recommend IVIG (2 g/kg) combined with a bolus of methylprednisolone (30 mg/kg/day) and antiplatelet aspirin (3–5 mg/kg/day) (Figure 3). In low-risk patients, in cases of persistent fever, a second infusion of IVIG (2 g/kg) should be administered alongside aspirin (30–50 mg/kg/day) (Figure 3) (117). If the fever remains unresponsive, we recommend methylprednisolone (30 mg/kg/day) administration for three consecutive days, and then daily prednisone (2 mg/kg/day, with tapering) and continued anti-aggregating aspirin (3–5 mg/kg/day) (Figure 3).

In high-risk or IVIG-resistant cases, corticosteroids play a pivotal role. Our clinical experience demonstrates that timely corticosteroid use in high-risk patients reduces fever duration and halts coronary aneurysm progression. In these patients in second-line treatment, we recommend a second infusion of IVIG (2 g/kg) and methylprednisolone (30 mg/kg/day) administration for three consecutive days and then daily prednisone (2 mg/kg/day, with tapering), and continued anti-aggregating aspirin (3–5 mg/kg/day) (Figure 3). In persistent cases, infliximab (5 mg/kg) and anakinra (4–8 mg/kg/day for at least 15 days, SC) remain effective escalation options (Figure 3). Early risk stratification and monitoring using algorithms, such as CEDi and echocardiography, are invaluable for guiding management. The treatment of MAS in patients with KD should follow the same principles as in Still's disease.

#### 7.2.3 Multisystem inflammatory syndrome in children

MIS-C presents a diagnostic challenge for the physician due to the nonspecific early symptoms, lack of pathognomonic findings, and potentially fatal complications (149). WHO case definitions are the best criteria for diagnosis of MIS-C patients (149, 150).

**Figure 4:**
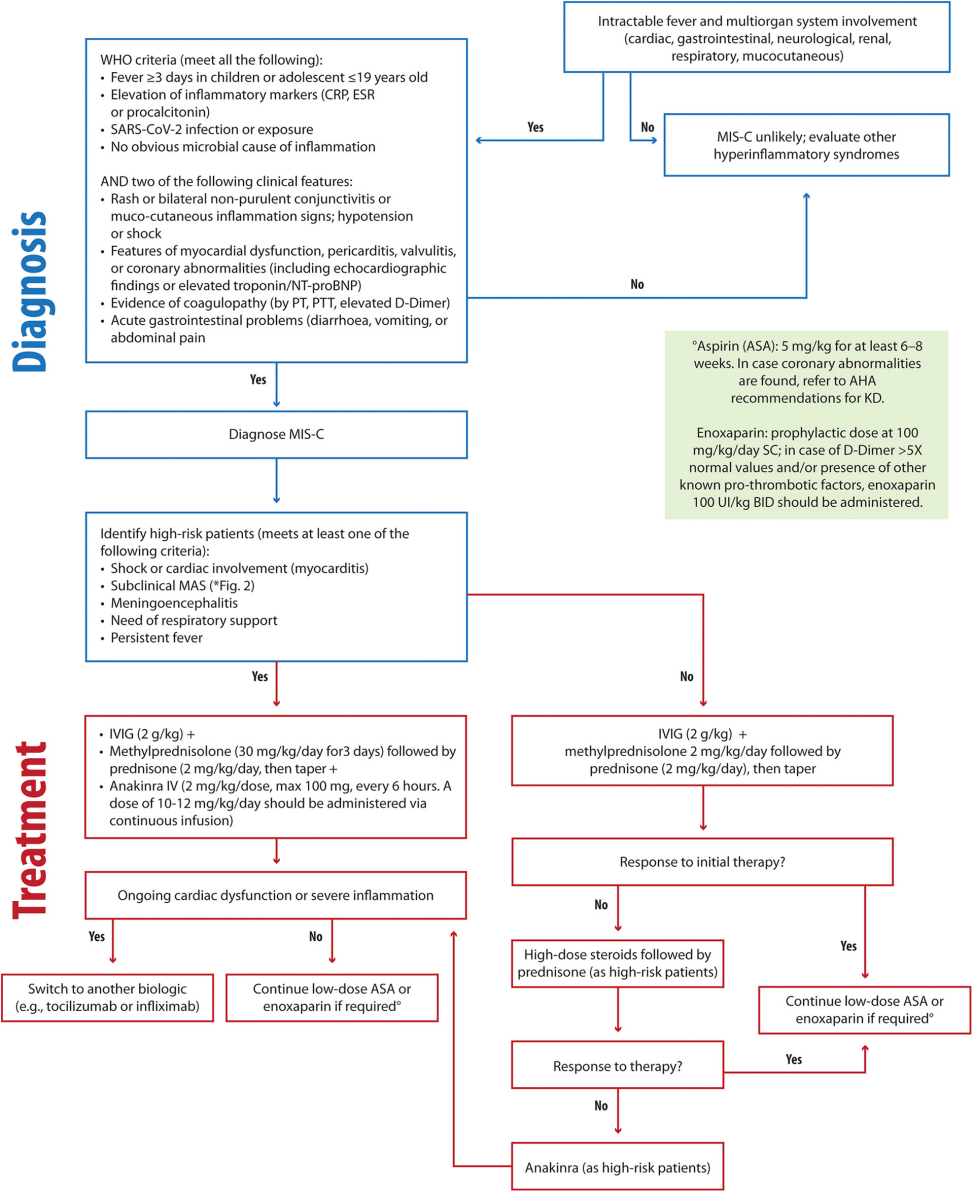
Clinical flowchart illustrating the diagnostic and therapeutic approach to MIS-C. MIS-C, Multisystem Inflammatory Syndrome in Children; CRP, C-reactive protein; ESR, Erythrocyte Sedimentation Rate; NT-proBNP, N-terminal pro-B-type natriuretic peptide; PT, prothrombin time; PTT, partial thromboplastin time; MAS, macrophage activation syndrome; AHA, Americal Heart Association; IVIG, intravenous immunoglobulin; ASA, acetylsalicylic acid.

A study by La Torre et al. (148) evaluated 22 MIS-C patients with a mean age of 7.4 years in the Apulia region over a period of 6 months spanning from 1 November 2020 to 30 April 2021. The most prevalent symptoms were gastrointestinal involvement, cardiovascular involvement, features of KD, and MAS/HLH (present in 23% of cases). No patients were admitted to the Pediatric Intensive Care Unit (PICU) in this study (148). In severe presentations, treatment included IVIG infusions and methylprednisolone boluses (148). In cases of MAS, shock, myocarditis, or meningoencephalitis, endovenous anakinra (2 mg/kg, max 100 mg, every 6 h) was utilized as a first-line therapy (148). Combining high-dose steroids with anakinra demonstrated efficacy in restoring myocardial function, particularly in patients with MAS-like features or significant cardiac involvement, reducing the rates of admission to PICU (Figure 4). In our opinion, the rapid and flexible application of anakinra is noteworthy, as it is suited for “hybrid” conditions requiring immediate intervention. In fact, the prompt step-up treatment with IVIG, steroids, and anakinra, depending on the organ involvement or response to therapy, contributed to good outcomes in terms of PICU admission and long-term cardiac sequelae (148, 171).

To optimize care, we emphasize the importance of equipping cardiologists with specialized pediatric cardiology training and establishing dedicated registries to collect data from larger patient cohorts is essential for developing robust clinical guidelines.

#### 7.2.4 Recurrent pericarditis

There are no clear differences regarding clinical presentation between acute and recurrent pericarditis. However, a symptom-free interval of 4–6 weeks and evidence of new pericardial inflammation are needed for the diagnosis (201). Effective treatment of acute pericarditis significantly reduces the risk of recurrence (201).

Colchicine is commonly employed in pericarditis management (28, 30, 184). Three studies are shown supporting the efficacy of colchicine in recurrences (202–204). A multicenter, double-blind study by Imazio et al. (194) demonstrated a reduced incidence of post-pericardiotomy syndrome with colchicine prophylaxis in surgical patients. However, due to a high incidence of adverse effects, routine colchicine use is not recommended for all surgical patients (205). IL-1 blockade with anakinra is beneficial for the treatment of recurrent pericarditis (184, 187, 188), as shown by several case series (206–208), the IRAP study (209) and the randomized controlled AIRTRIP (Anakinra-Treatment of Recurrent Idiopathic Pericarditis) trial (188).

**Figure 5:**
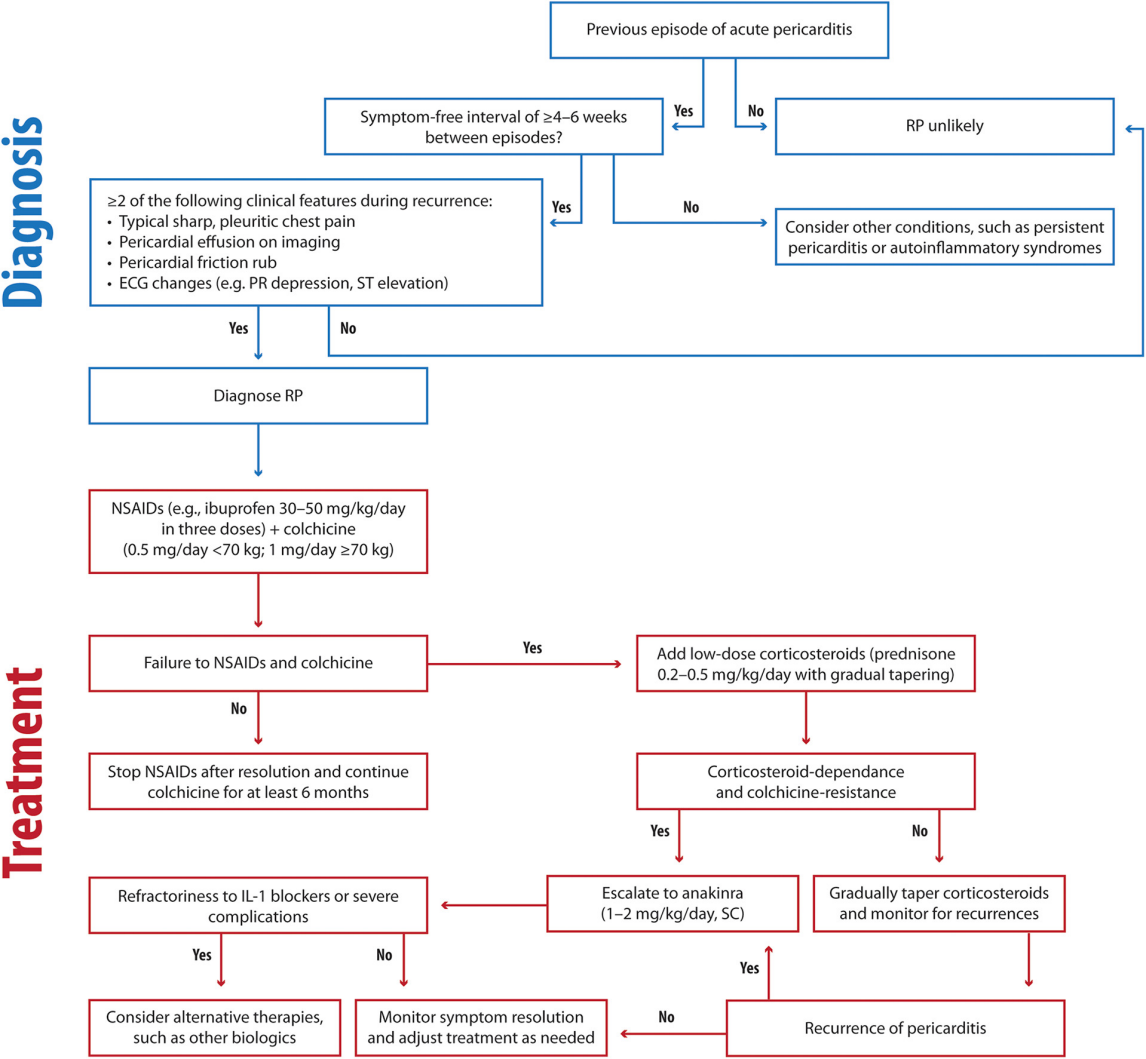
Clinical flowchart illustrating the diagnostic and therapeutic approach to RP. RP, recurrent pericarditis; ECG, electrocardiography; NSAIDs, Nonsteroidal anti-inflammatory drugs; SC: subcutaneous.

For treating RP, we recommend the following approach (Figure 5):

- First-line therapy
  - NSAIDs, including ibuprofen (30–50 mg/kg/day), aspirin (30–50 mg/kg/day), and colchicine (0.5 mg/day for children under 70 kg, 1 mg/day for children over 70 kg)
- Second-line therapy
  - Steroids, particularly for cases unresponsive to NSAIDs and colchicine. Low-dose prednisone (0.2–0.5 mg/kg/day) is more effective in reducing recurrences, hospitalization duration, and side effects.
- Third-line therapy
  - Anakinra 1–2 mg/kg/day, max 100 mg, SC) is recommended as a steroid-sparing option, with prolonged use proving effective in preventing relapses, even in refractory cases.

Comprehensive combination therapy further reduces recurrence risk while restricting intense physical activity until symptom resolution, guided by inflammatory markers, is essential.

The long-term prognosis for idiopathic RP is generally favorable, with cardiac tamponade and constrictive pericarditis remaining rare.

### 7.3 Open questions and unmet needs

Multidisciplinary collaboration among rheumatologists, cardiologists, and intensivists is essential, particularly in high-risk cases requiring complex, coordinated care. Despite advancements, several unresolved questions and challenges remain in the management of Still's disease. Key issues include the lack of predictive factors for therapeutic outcomes, uncertainty about the respective indications for IL-1 and IL-6 inhibitors, and the absence of consensus on tailoring treatment based on the presence or absence of arthritis. Diverging opinions on corticosteroid use, limited options for cases resistant to IL-1 and IL-6 inhibitors, and the lack of standardized protocols for tapering or discontinuing treatment further complicate management.

Many of these challenges also apply to conditions like KD, MIS-C, and RP. The lack of reliable predictive biomarkers hinders the precision of treatment approaches. Biomarkers that guide responses to IL-1 and IL-6 inhibitors are urgently needed. Advances in genetic testing and immune profiling hold potential for personalized treatment strategies but require further validation and integration into routine clinical care.

Anakinra has emerged as a promising option for reducing corticosteroid dependency across multiple inflammatory conditions, including Still's disease and RP. Challenges persist in managing colchicine-resistant and steroid-dependent RP cases, mitigating corticosteroid side effects, and addressing the impact of recurrences on quality of life. Anakinra provides a viable steroid-sparing alternative. However, head-to-head trials comparing anakinra and corticosteroids in pediatric populations are needed to establish optimal dosing and duration. Establishing effective strategies for such cases and enhancing long-term management approaches remain key areas for future research.

Similarly, the absence of consensus on tapering or withdrawing biologics complicates the long-term management of conditions, such as KD, MIS-C, and RP. Premature withdrawal may lead to disease flares, while prolonged use increases costs and the risk of side effects. Longitudinal studies are necessary to define safe and effective withdrawal protocols.

Additionally, patients unresponsive to IL-1 or IL-6 inhibitors represent a significant treatment gap.

Novel therapies, such as JAK inhibitors for Still's diseaseand biologics targeting IFN-*γ* or IL-18, show potential for refractory cases but require further research to confirm efficacy and safety.

Further research is needed to understand more in-depth the mechanisms of immune dysregulation underlying these conditions, along with the impact of genetic predispositions and environmental triggers.

Finally, as mentioned above, regional training programs in pediatric cardiology are urgently needed to address gaps in expertise. Conditions like MIS-C and KD underscore the importance of equipping centers with specialists skilled in managing inflammatory and autoimmune conditions. Such initiatives can enhance timely and accurate interventions, ultimately improving patient outcomes.

## 8 Conclusion

Despite advances in Still's disease management, challenges persist due to clinical variability, risk of complications, and the need for early intervention. Research into biomarkers, novel therapies, and personalized approaches remains vital to improving outcomes.

Hyperinflammatory diseases, such as Still's disease, KD, MIS-C, and RP, share overlapping features but differ in pathophysiology and clinical courses. Key findings include:

- Still's disease: IL-1 or IL-6 inhibitors as first-line therapy improve outcomes, guided by biomarkers like ferritin and sJADAS.
- KD: IVIG and aspirin prevent coronary complications; corticosteroids and anakinra aid in high-risk cases. Tools like CEDi enhance risk stratification.
- MIS-C: IVIG, corticosteroids, and anakinra reduce mortality, with biomarkers like NT-proBNP aiding diagnosis and management of cardiac involvement.
- RP: NSAIDs and colchicine remain foundational, while anakinra addresses colchicine-resistant cases, minimizing recurrence and corticosteroid use.

The integration of genetic testing and biomarker-guided treatments, such as serum calprotectin in Still's diseaseor NT-proBNP in MIS-C, offers precision in identifying complications like MAS and myocarditis. Imaging advances, including cardiac MRI, further enhance diagnostic and monitoring capabilities.

Future research should focus on immune dysregulation mechanisms and trials comparing existing and novel therapies, such as anakinra vs. corticosteroids or JAK inhibitors for refractory cases. Long-term management would benefit from safe withdrawal protocols for biologics to prevent flares while minimizing costs and side effects.

Developing regional expertise in pediatric inflammatory diseases through training programs will ensure timely, specialized care and improve outcomes. Addressing these research and clinical gaps will advance personalized medicine, reducing complications and enhancing quality of life.
